# *In vitro* and *in vivo* effects of Galectin‐3 inhibitor TD139 on inflammation and ERK/JNK/p38 pathway in gestational diabetes mellitus

**DOI:** 10.1002/kjm2.12890

**Published:** 2024-09-04

**Authors:** Ji Xia, Yan Wang, Bang‐Ruo Qi

**Affiliations:** ^1^ Department of Obstetrics, Hainan Branch, Shanghai Children's Medical Center, School of Medicine Shanghai Jiao Tong University Sanya China

**Keywords:** Galectin 3, gestational diabetes, inflammation, mitogen‐activated protein kinases (MAPKs), tumor necrosis factor‐alpha (TNF‐α)

## Abstract

This study aims to investigate the effects of the Galectin‐3 (Gal‐3) inhibitor TD139 on inflammation and the extracellular signal‐regulated kinase (ERK)/c‐Jun N‐terminal kinase (JNK)/p38 pathway in gestational diabetes mellitus (GDM). Human placental tissues were treated with TD139 and TNF‐α, assessing Gal‐3, ERK/JNK/p38 activation, and inflammatory cytokines. GDM was induced in mice via subcutaneous injections of streptozotocin (STZ). After confirming GDM, mice were treated with 15 mg/kg TD139 on GD 10.5 12.5, 14.5, 16.5, and 18.5. Serum inflammatory cytokines were measured on GD 20.5, and post‐delivery placental tissues were analyzed. Data were analyzed using one‐way or two‐way repeated measures ANOVA with post hoc tests. TD139 suppressed TNF‐α‐induced increases in Gal‐3, IL‐1β, IL‐6, MCP‐1, and ERK/JNK/p38 activation in placental tissues. In STZ‐induced GDM mice, TD139 reduced glucose levels, weight loss, and food and water intake. TD139 significantly lowered TNF‐α, IL‐1β, IL‐6, and MCP‐1 in serum and placental tissues and inhibited the ERK/JNK/p38 pathway. TD139 improved pup numbers in GDM mice compared to untreated ones. TD139 reduces inflammation and inhibits the ERK/JNK/p38 pathway in TNF‐α stimulated placental tissues and STZ‐induced GDM mice, suggesting its therapeutic potential for managing GDM‐related placental inflammation and improving pregnancy outcomes. The study used TNF‐α to mimic GDM in placental tissues and an STZ‐induced GDM mouse model, which may not fully represent human GDM complexity. Future research should explore alternative models, and broader signaling pathways, and thoroughly evaluate TD139's safety in pregnancy.

## 1 INTRODUCTION

During pregnancy, diabetes mellitus may manifest as pre‐existing type 1 diabetes mellitus (T1DM) or type 2 diabetes mellitus (T2DM), or gestational diabetes mellitus (GDM), with glucose metabolic abnormalities classified as pre‐existing diabetes complicating pregnancies, evident diabetes manifesting during pregnancy, or GDM in humans.^1^ Over the past four decades, the global prevalence of GDM has steadily increased, with Southeast Asia, including China, having the highest median prevalence at 25.9%.^2^ Inadequately managed GDM heightens the likelihood of severe short‐ and long‐term complications for both the mother and the child.^3^ Therefore, elucidating the mechanisms underlying GDM is crucial for improving maternal and fetal health.

Galectin‐3 (Gal‐3), formerly known as Mac‐2, L‐29, L‐31, L‐34, IgE binding‐protein, CBP35, and CBP30, is a monomer in its basal state, but at higher concentrations, it can assemble into multimers (dimers or pentamers).^4^ In recent years, elevated circulating levels of Gal‐3 have been proposed as biomarkers for guiding liraglutide therapy in T2DM patients.^5^ Gal‐3 contributed to beta‐cell apoptosis via the mitochondrial pathway under inflammatory conditions, suggesting it is a promising therapeutic target for diabetes.^6^ The extracellular signal‐regulated kinase (ERK)/c‐Jun N‐terminal kinase (JNK)/p38 pathway signaling pathway is integral to cellular responses to stress, inflammation, and other stimuli, and its activation has been linked to diabetes and its complications.^7^ Gal‐3 modulates diverse pathological processes such as cardiac ischemia–reperfusion injury and ultraviolet‐induced skin inflammation by influencing the JNK pathway and phosphorylation of p38, respectively, while in neuroblastoma, Gal‐3BP activates IL‐6 production in mesenchymal stem cells through the ERK signaling pathway.^8^, ^9^, ^10^

Selective galectin inhibitors such as modified citrus pectin, N‐acetyllactosamine, TD139, and GB0139 exert anti‐apoptotic, anti‐oxidative, and anti‐inflammatory effects, which are valuable research tools and could also be used as drug candidates.^11^ In that context, TD139, a high‐affinity Gal‐3 inhibitor targeting the CRD, has demonstrated safety and tolerability in clinical trials involving healthy individuals and patients with idiopathic pulmonary fibrosis.^12^ According to previous studies, Gal‐3 inhibitors such as TD139 could reduce Gal‐3 expression, potentially improving diabetes and its complications.^13^ The current literature has established a connection between elevated Gal‐3 levels and GDM, suggesting that Gal‐3 may play a role in the pathophysiology of this condition. Studies by Zhang et al. and Talmor‐Barkan et al. have indicated that Gal‐3 levels are elevated in GDM,^14^, ^15^ and Heusler et al. have reported altered Gal‐3 expression in GDM placental tissue.^16^ These findings suggest that Gal‐3 might contribute to GDM‐related complications and could potentially serve as a biomarker for the disease. Despite these associations, there is a significant lack of research on the effects of Gal‐3 inhibitors on GDM. This gap highlights the need for studies to assess whether targeting Gal‐3 with inhibitors, such as TD139, could influence disease progression or improve outcomes in GDM. Our study seeks to address this research gap by investigating the potential benefits of Gal‐3 inhibition using TD139 in the context of GDM. This could advance our understanding of Gal‐3's role in GDM and explore new therapeutic strategies for managing the condition.

This study aims to investigate the effects of TD139 on inflammation and the ERK/JNK/p38 pathway in GDM. In vitro, we treated human placental tissues with TD139 and assessed changes in Gal‐3 expression, ERK/JNK/p38 signaling pathway activation, and inflammatory cytokine levels after stimulation with tumor necrosis factor‐alpha (TNF‐α). *In vivo*, we induced GDM in mice using streptozotocin (STZ) and treated them with TD139. We then monitored glucose levels, body weight, food and water intake, and inflammatory cytokine expression in both serum and placental tissues. Our study aims to provide insights into the potential therapeutic benefits of TD139 in managing GDM‐related inflammation and improving pregnancy outcomes.

## 2 METHODS AND MATERIALS

### 2.1 Ethics statement

This study received ethics approval from our hospital. Informed consent was obtained from all participants involved in the study, ensuring their full awareness and consent for the collection of human placental tissues.

### 2.2 In vitro experiments

Placental tissues were obtained from six healthy women with normal glucose tolerance, aged 31.17 ± 3.1 years, and having a body mass index <30 kg/m^2^. These women delivered healthy, singleton infants at term (37–41 weeks of gestation) via elective cesarean section without labor. Following delivery, placentas were promptly collected, processed, and immersed in PBS at 4°C. After removing visible connective tissue and calcium deposits, the tissues were washed thoroughly in PBS and pre‐incubated in Dulbecco's Modified Eagle's Medium supplemented with 100 U/mL penicillin G and 100 μg/mL streptomycin at 37°C in a humidified atmosphere containing 5% CO_2_ and 8% O_2_. Subsequently, 100 mg wet‐weight placental samples were incubated in 1 mL DMEM for 20 h. The tissues were then treated overnight with or without 10 μM TD139 (S0471, Selleck, China),^17^ followed by a 30‐min incubation with TNF‐α (10 ng/mL) to establish GDM‐like environment.^18^ Control samples comprised untreated human placental tissues.

### 2.3 Animal grouping

The experimental design involved four groups: Control + vehicle, Control + TD139, STZ + vehicle, and STZ + TD139 groups. To determine the appropriate sample size, we used the formula proposed by Arifin et al.^19^ for group comparison (ANOVA) with degrees of freedom (DF) ranging from 10 to 20: N = (DF/κ) + 1, where κ is the number of groups.^20^ This calculation indicated a maximum requirement of 6 animals per group. Consequently, the study utilized twenty‐four 7‐week‐old female CD‐1® Nude Mice (Charles River Laboratories, USA), raised under pathogen‐free conditions. The mice were housed in two per cage, fed a standard diet (Rodent Laboratory Chow 5001 from Purina, 3.02 Kcal/g), and given ad libitum access to water. Environmental conditions were maintained at 19–21°C with a 12‐h light/dark cycle.

### 2.4 Induction of GDM and TD139 treatment

**FIGURE 1:**
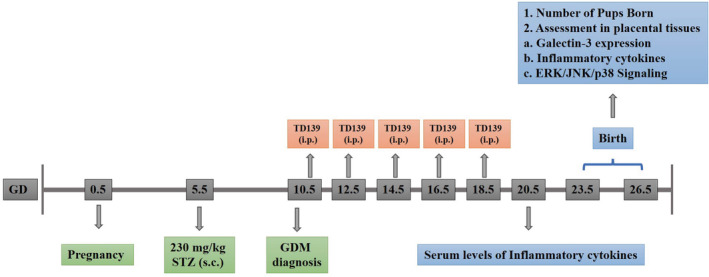
Experimental design *in vivo*. TD139, a Galectin‐3 (Gal‐3) inhibitor, was administered by intraperitoneal injection (15 mg/kg, i.p.) on gestational days (GD) 10.5, 12.5, 14.5, 16.5, and 18.5 after the diagnosis of gestational diabetes mellitus (GDM) induced by subcutaneous (s.c.) administration of 230 mg/kg streptozotocin (STZ).

Figure 1 outlines the experimental timeline. GDM induction coincided with pregnancy onset. Five days prior to GDM induction using STZ, two females were mated with one male. Confirmation of pregnancy was marked by the presence of a vaginal plug, indicating gestation day 0.5 (GD 0.5). On GD 5.5, after pregnancy confirmation, pregnant mice received subcutaneous injections of 230 mg/kg STZ (Cat. No. S0130, Merck, China)^21^ to induce GDM. Postprandial blood glucose levels were monitored for five subsequent days. GDM diagnosis was confirmed on GD 10.5, adhering to the criteria set by the American Diabetes Association (ADA) in 2018, including sustained glucose levels ≥200 mg/dL, and clinical symptoms like polyphagia, polydipsia, and weight changes.^22^ Following GDM confirmation, mice received intraperitoneal injections of either dimethyl sulfoxide (DMSO) as a control or 15 mg/kg TD139 in DMSO^23^ on GD 10.5, 12.5, 14.5, 16.5, and 18.5. Animal weights were measured using an Ohaus™ Triple Beam 700/800 Series mouse scale, while food intake was determined by weighing the remaining feed daily using an Ohaus™ CS series Model CS200‐001 scale, and water consumption was measured using a 50 mL test tube. Upon delivery, mice were euthanized by cervical dislocation, and placental tissues were promptly collected, flash‐frozen in liquid nitrogen, and stored at −80°C until Quantitative reverse transcription polymerase chain reaction (qRT‐PCR) and Western blot analysis. Careful removal of the decidua was performed to prevent maternal tissue contamination.

### 2.5 Cytokine analysis

The expression levels of inflammatory cytokines in placental tissues and serum, including TNF‐α, interleukin‐1β (IL‐1β), interleukin‐6 (IL‐6), and monocyte chemoattractant protein‐1 (MCP‐1), were measured using RT‐qPCR analysis and enzyme‐linked immunosorbent assay (ELISA) kits. ELISA kits were purchased from Thermo Fisher Scientific Inc. (Shanghai, China).

### 2.6 Quantitative reverse transcription polymerase chain reaction

Total RNA was isolated using the TRIzol™ Plus RNA Purification Kit according to the manufacturer’ s protocol (Catalog No.12183555CN, Thermo Fisher Scientific Inc., USA). Briefly, samples were lysed with TRIzol reagent, which maintains RNA integrity and inhibits RNase activity. The lysed samples were then applied to the PureLink RNA Mini Kit Spin Cartridge for RNA binding, followed by washing to remove contaminants, and elution in RNase‐free water or Tris buffer (pH 7.5). Quantitative RT‐PCR was performed using the SuperScript III Platinum One‐Step Quantitative RT‐PCR System with ROX (Catalog No. 11745500, Thermo Fisher Scientific Inc., USA) on an ABI 7300 real‐time PCR System. Efficient cDNA synthesis can be accomplished in a 15‐min incubation at 50°C. Gene‐specific primers are listed in Table 1. All samples were analyzed in duplicate and normalized to the housekeeping gene GAPDH.

**TABLE 1:** Primers used in quantitative RT‐PCR.

Gene	Species	GenBank accession	Primers sequence (5′ → 3′)
Interleukin 1 beta (Il1b)	Mouse	NM_008361	Forward: GCAACTGTTCCTGAACTCAACT
			Reverse: ATCTTTTGGGGTCCGTCAACT
Interleukin 6 (Il6)	Mouse	NM_031168	Forward: TAGTCCTTCCTACCCCAATTTCC
			Reverse: TTGGTCCTTAGCCACTCCTTC
Monocyte chemoattractant protein‐1 (MCP‐1)	Mouse	NM_011333	Forward: TTAAAAACCTGGATCGGAACCAA
			Reverse: GCATTAGCTTCAGATTTACGGGT
Glyceraldehyde‐3‐phosphate dehydrogenase (Gapdh)	Mouse	NM_001289726	Forward: AGGTCGGTGTGAACGGATTTG
			Reverse: TGTAGACCATGTAGTTGAGGTCA
Galectin‐3 (Gal‐3)	Human	NM_001177388	Forward: ATGGCAGACAATTTTTCGCTCC
			Reverse: GCCTGTCCAGGATAAGCCC
IL‐1β	Human	NM_000576	Forward: ATGATGGCTTATTACAGTGGCAA
			Reverse: GTCGGAGATTCGTAGCTGGA
IL‐6	Human	NM_000600	Forward: ACTCACCTCTTCAGAACGAATTG
			Reverse: CCATCTTTGGAAGGTTCAGGTTG
MCP‐1	Human	NM_002982	Forward: CAGCCAGATGCAATCAATGCC
			Reverse: TGGAATCCTGAACCCACTTCT
GAPDH	Human	NM_001256799	Forward: ACAACTTTGGTATCGTGGAAGG
			Reverse: GCCATCACGCCACAGTTTC

### 2.7 Western blotting

Placental tissues were lysed in a buffer containing protease inhibitors and homogenized on ice. After centrifugation, the protein concentration in the supernatant was determined using a bicinchoninic acid protein assay (Catalog No. A65453, Thermo Scientific, Waltham, MA, USA) and stored at −80°C. Equal amounts of total protein from each group were separated by electrophoresis on a 15% SDS‐PAGE (Catalog No. 89888, Thermo Scientific, Waltham, MA, USA) and transferred to a PVDF membrane (Catalog No. 24585, Thermo Scientific, Waltham, MA, USA). The membranes were blocked with 3% tris‐buffered saline containing 0.5% (v/v) Tween 20 (TBST)‐milk for 1 h, washed three times with TBS buffer, and then incubated overnight at 4°C with primary antibodies. The primary antibodies used were: anti‐Gal‐3 antibody at 1/10000 dilution (ab76245), anti‐phospho ERK antibody at 1/1000 dilution (ab201015), anti‐ERK antibody at 1/10000 dilution (ab184699), anti‐phospho JNK antibody at 1/1000 dilution (ab4821), anti‐JNK antibody at 1/1000 dilution (ab112501), anti‐phospho p38 antibody at 1/1000 dilution (ab195049), anti‐p38 antibody at 1/5000 dilution (ab170099), and anti‐β‐actin antibody as a loading control at 1 μg/mL (ab8226). After washing, the membranes were incubated with goat anti‐rabbit IgG H&L (HRP) at 1/1000 dilution (ab97051). Immunoreactive bands were detected using chemiluminescence (Catalog No. 32106, Thermo Scientific, Waltham, MA, USA), and densitometry analysis was performed using ImageJ software (NIH, Wayne Rasband, USA).

### 2.8 Statistical analysis

Data were represented as the mean ± SD. First, we performed the Kolmogorov–Smirnov normality test to determine whether the data distribution was parametric or nonparametric. Either a one‐way or two‐way repeated measures ANOVA followed by a post hoc test was run when appropriate. Statistical significance was accepted at *p* < 0.05. All statistical analyses were performed using GraphPad Prism version 8.0 software (San Diego, CA, United States).

## 3 RESULTS

### 3.1 TD139 suppresses TNF‐α induced inflammation and inhibits ERK/JNK/p38 signaling in human placental tissues *in vitro*

**FIGURE 2:**
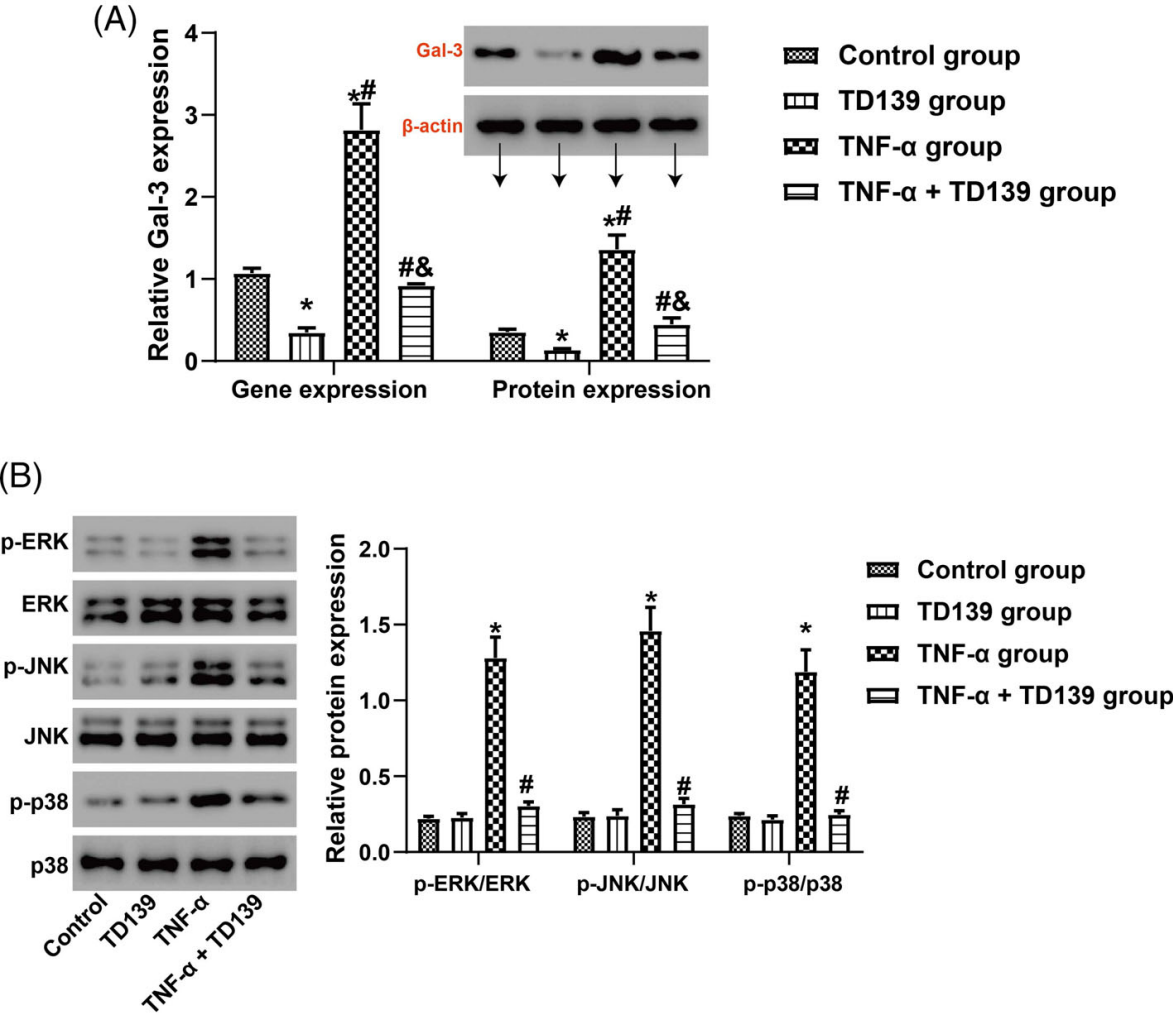
TD139 inhibited the activation of the ERK/JNK/p38 signaling pathway in TNF‐α stimulated placenta *in vitro*. Human placental tissues were incubated overnight with or without 10 μM TD139, followed by a 30‐min incubation with tumor necrosis factor‐alpha (TNF‐α, 10 ng/mL). (a) Both quantitative reverse transcription polymerase chain reaction (qRT‐PCR) and Western blotting were employed to assess Galectin‐3 (Gal‐3) gene and protein expression in the different groups; * indicates *p<*0.05 compared to the Control group, # indicates *p<*0.05 compared to the TD139 group, & indicates *p<*0.05 compared to the TNF‐α group. *n* = 6. (b) Western blotting was used to evaluate the expression of key proteins in the mitogen‐activated protein kinase (MAPK) pathway, including extracellular signal‐regulated kinase (ERK), c‐Jun N‐terminal Kinase (JNK), and p38. * indicates *p<*0.05 compared to the Control group and the TD139 group, # indicates *p<*0.05 compared to the TNF‐α group. *n* = 6.

**FIGURE 3:**
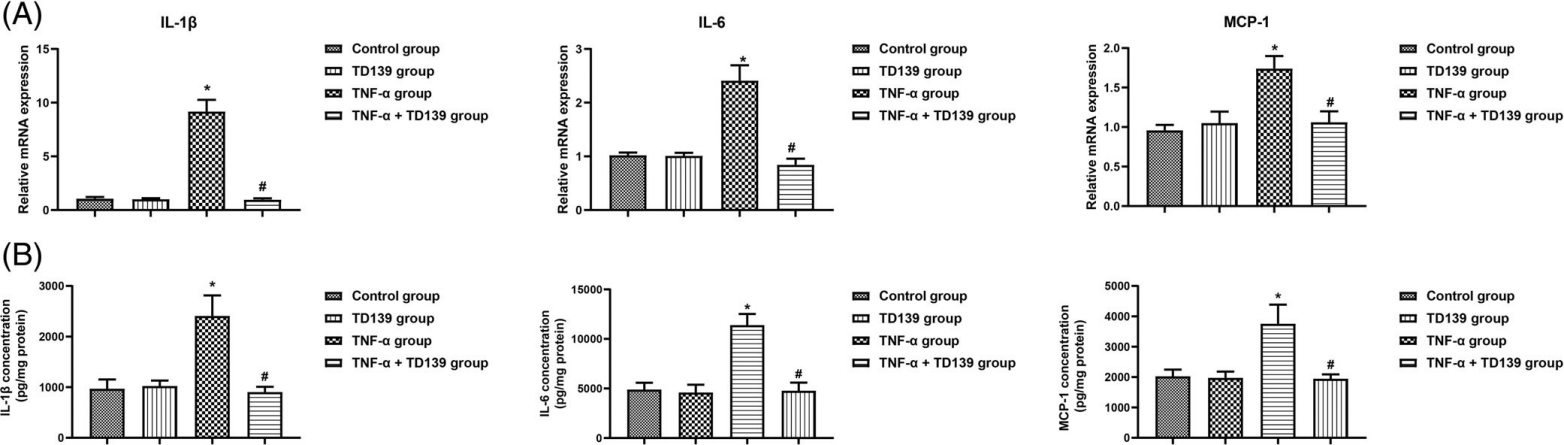
TD139 decreased TNF‐α stimulated inflammation in placenta in vitro. Human placental tissues were incubated overnight with or without 10 μM TD139, followed by a 30‐min incubation with tumor necrosis factor‐alpha (TNF‐α, 10 ng/mL). The expression of inflammatory cytokines interleukin‐1 beta (IL‐1β), interleukin‐6 (IL‐6), and monocyte chemoattractant protein‐1 (MCP‐1) was analyzed by Reverse transcription‐quantitative polymerase chain reaction (RT‐qPCR) analysis (A) and enzyme‐linked immunosorbent assay (ELISA) (b). * indicates *p<*0.05 compared to the Control group and the TD139 group, # indicates *p<*0.05 compared to the TNF‐α group. *n* = 6.

Human placental tissues were incubated overnight with or without 10 μM TD139, followed by a 30‐min incubation with TNF‐α (10 ng/mL). Both RT‐qPCR and Western blotting were employed to assess Gal‐3 gene and protein expression across different groups. The results indicated that TD139 treatment alone decreased Gal‐3 expression. TNF‐α stimulation significantly increased Gal‐3 expression at both the gene and protein levels (all *p <*0.05), which was significantly suppressed by TD139 treatment (all *p <*0.05, Figure 2A). Western blotting was utilized to assess the expression of the ERK/JNK/p38 signaling pathway. TD139 treatment alone did not affect the ERK/JNK/p38 signaling pathway in human placental tissues. However, TNF‐α stimulation activated this pathway, as evidenced by increased levels of phosphorylated ERK (p‐ERK/ERK), JNK (p‐JNK/JNK), and p38 (p‐p38/p38) (all *p*<0.05). TD139 effectively reversed this activation, significantly downregulating the expression of phosphorylated ERK/JNK/p38 pathway proteins (all *p*<0.05, Figure 2B). qPCR analysis (Figure 3A) revealed that compared to the control group and TD139 group, TNF‐α significantly upregulated the expression of inflammatory cytokines IL‐1β, IL‐6, and MCP‐1 (all *p<*0.05). However, co‐treatment with TD139 significantly downregulated the expression of these inflammatory cytokines (all *p<*0.05). ELISA measurements of these inflammatory cytokine protein levels in placental tissues showed results consistent with the gene expression levels (Figure 3B).

### 3.2 Effects of TD139 on glucose, weight, food, and water consumption in STZ‐induced GDM mice *in vivo*

Table 2 presents a comparison of glucose levels, body weight, food intake, and water consumption among different groups of GDM mice on GD 0.5 (indicative of pregnancy), GD 5.5 (before STZ injection), on GD 10.5 (GDM diagnosis and prior to TD139 treatment), on GD 15.5, on GD 20.5 and GD 23 to 26 (post‐birth). Initial glucose levels (GD 0.5 and GD 5.5) were consistent across all groups. However, from GD 10.5 onwards, a notable increase in glucose concentration was observed in the STZ + vehicle group (*p*<0.05), indicating successful GDM induction. In contrast, the STZ + TD139 group exhibited significantly reduced glucose levels post‐TD139 treatment (*p* < 0.05), highlighting the efficacy of TD139 in mitigating hyperglycemia. Initial body weights, food consumption, and water consumption were similar across experimental groups (all *p* > 0.05). However, from GD 10.5 onwards, the STZ + vehicle group experienced significant weight reduction, accompanied by a notable increase in food intake suggestive of polyphagia, and a marked surge in water consumption indicative of polydipsia (*p*<0.05). In contrast, the STZ + TD139 group demonstrated reduced weight loss compared to the STZ + vehicle group, as well as decreased food consumption and significantly decreased water intake (*p*<0.05).

**TABLE 2:** Comparison of glucose levels, body weight, food intake, and water consumption across different groups.

	Control + vehicle group (*n* = 6)	Control + TD139 group (*n* = 6)	STZ + vehicle group (*n* = 6)	STZ + TD139 group (*n* = 6)
Glucose concentration (mg/dL)				
GD 0.5	134 ± 17	113 ± 22	129 ± 16	120 ± 23
GD 5.5	117 ± 21	117 ± 21	118 ± 24	117 ± 12
GD 10.5	118 ± 22	117 ± 13	377 ± 19 *	366 ± 21 *
GD 15.5	123 ± 13	130 ± 19	483 ± 13 *	255 ± 17 ^*#^
GD 20.5	133 ± 17	122 ± 20	505 ± 13 *	205 ± 17 ^*#^
GD 23–26	122 ± 21	120 ± 15	537 ± 12 *	196 ± 14 ^*#^
Weight (g)				
GD 0.5	27.3 ± 0.7	27.7 ± 0.7	26.5 ± 0.7	26.5 ± 0.7
GD 5.5	27.7 ± 0.8	27.5 ± 0.7	26.1 ± 0.8	26.1 ± 0.8
GD 10.5	28.9 ± 0.8	28.0 ± 0.8	31.9 ± 0.8 *	31.9 ± 0.8 *
GD 15.5	38.7 ± 1.1	39.2 ± 0.8	32.8 ± 1.4 *	35.8 ± 2.2 ^*#^
GD 20.5	47.9 ± 1.0	46.4 ± 1.0	34.3 ± 1.5 *	40.4 ± 1.5 ^*#^
GD 23–26	52.1 ± 1.9	51.6 ± 2.3	36.8 ± 3.2 *	42.5 ± 2.6 ^*#^
Food (g)				
GD 0.5	31.7 ± 0.2	33.0 ± 0.2	31.4 ± 0.2	33.7 ± 0.2
GD 5.5	22.0 ± 0.2	23.8 ± 0.2	24.0 ± 0.2	23.8 ± 0.2
GD 10.5	26.9 ± 0.8	28.0 ± 0.8	40.9 ± 8.3 *	40.0 ± 0.8 *
GD 15.5	26.1 ± 0.2	27.2 ± 0.8	40.6 ± 1.4 *	35.8 ± 2.5 ^*#^
GD 20.5	27.5 ± 0.2	29.6 ± 1.0	42.4 ± 1.5 *	35.3 ± 1.5 ^*#^
GD 23–26	27.4 ± 1.1	28.3 ± 1.1	44.4 ± 2.1 *	35.5 ± 1.7 ^*#^
Water (mL)				
GD 0.5	47.5 ± 2.9	51.0 ± 2.7	48.0 ± 3.3	49.0 ± 3.1
GD 5.5	50.5 ± 3.8	50.5 ± 3.1	51.5 ± 2.5	52.5 ± 3.7
GD 10.5	48.0 ± 3.7	49.5 ± 3.5	85.3 ± 3.7 *	82.7 ± 3.3 *
GD 15.5	50.5 ± 3.0	52.5 ± 3.1	91.4 ± 3.0 *	80.9 ± 3.2 ^*#^
GD 20.5	49.0 ± 3.2	52.0 ± 3.2	100.9 ± 2.9 *	87.2 ± 3.8 ^*#^
GD 23 26	52.5 ± 3.3	49.5 ± 2.8	104.0 ± 4.3 *	91.8 ± 4.2 ^*#^

*Note*: Gestational days (GD) 0.5 represents the time after mating and prior to inoculation with streptozotocin. GD 10.5 indicates the time after the confirmed diagnosis of GDM and prior to TD139 administration. GD 23 to 26 corresponds to the period after the birth of the pups. TD139 (15 mg/kg) was administered on GD 10.5, GD 12.5, GD 14.5, GD 16.5, and GD 18.5 via intraperitoneal (i.p.) injection. Asterisks (*) indicate *p*<0.05 compared to the Control + vehicle and Control + TD139 groups. Hash marks (#) indicate *p*<0.05 compared to the STZ + vehicle group.

### 3.3 Effects of TD139 on number of pups born in STZ‐induced GDM mice *in vivo*

The control groups (both with and without TD139) have a higher average number of pups born compared to the STZ‐induced GDM groups (both *p*<0.05). Specifically, the Control + vehicle group had a mean of 11.83 ± 1.602 pups, and the Control + TD139 group had a mean of 11.67 ± 1.033 pups. Notably, TD139 treatment in the STZ‐induced GDM group increased the mean number of pups to 9.667 ± 1.211 compared to the STZ + vehicle group, which had a mean of 7.667 ± 1.211 pups (*p* < 0.05).

### 3.4 TD139 suppresses inflammatory cytokines in serum and placenta of STZ‐induced GDM mice *in vivo*

**FIGURE 4:**
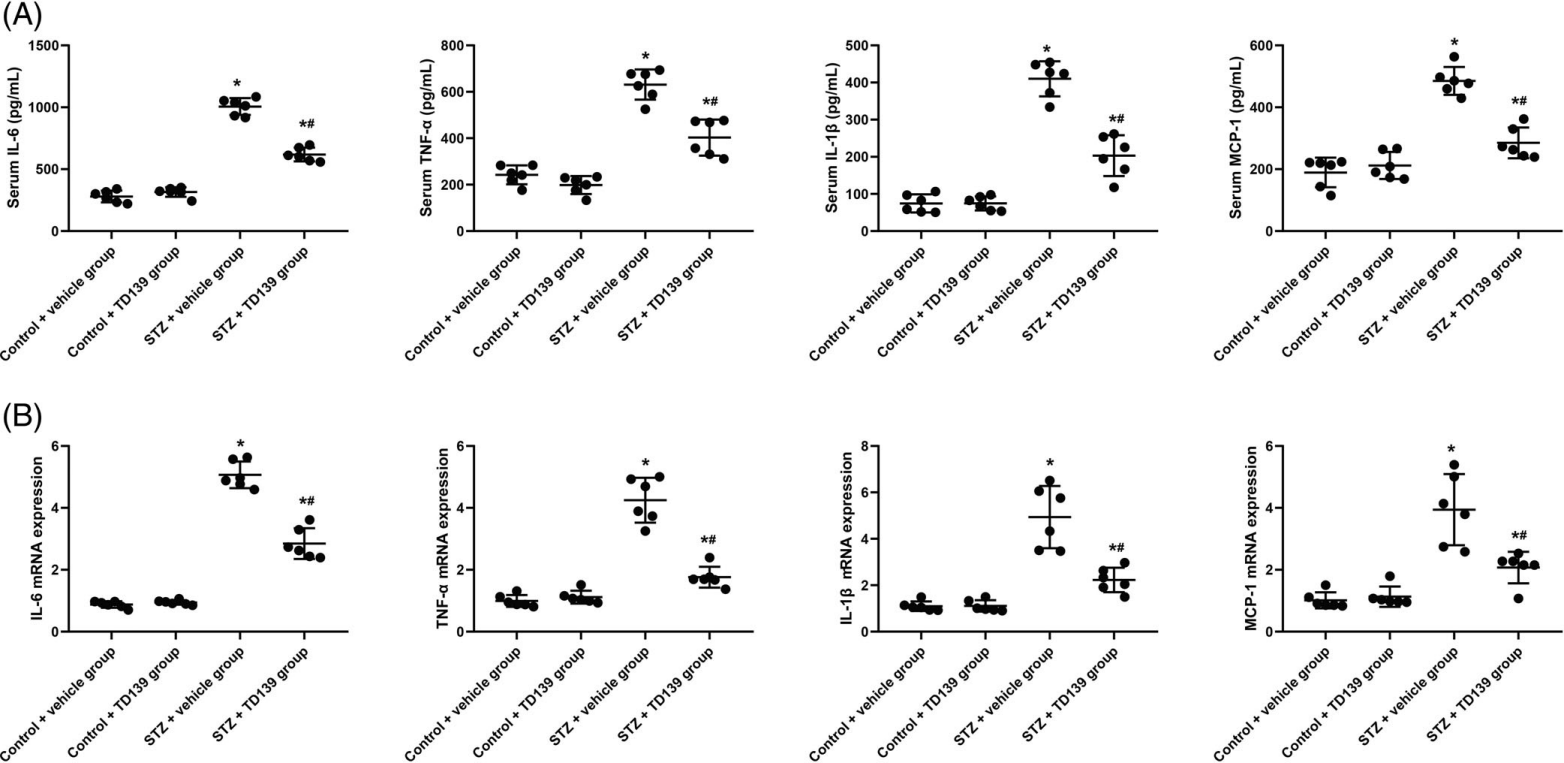
TD139 suppresses inflammatory cytokines in serum and placenta of streptozotocin (STZ)‐induced gestational diabetes mellitus (GDM) mice in vivo. The expression levels of inflammatory cytokines tumor necrosis factor‐alpha (TNF‐α), interleukin‐1 beta (IL‐1β), interleukin‐6 (IL‐6), and monocyte chemoattractant protein‐1 (MCP‐1) in the serum at gestational day 20.5 (measured using enzyme‐linked immunosorbent assay [ELISA], (a) and placental tissues after the birth of the pups (measured using quantitative reverse transcription polymerase chain reaction [qRT‐PCR], (b) of gestational diabetes mellitus (GDM) mice across different groups (*n* = 6 for each group). * indicates *p*<0.05 compared to the Control + vehicle group and the Control + TD139 group; # indicates *p*<0.05 compared to the STZ + vehicle group.

We assessed the expression levels of inflammatory cytokines TNF‐α, IL‐1β, IL‐6, and MCP‐1 in the serum on GD 20.5 (using ELISA, Figure 4A) and placental tissues after the birth of the pups (using RT‐qPCR, Figure 4B) of GDM mice across different groups. The findings revealed that the Control + vehicle group and the Control + TD139 group exhibited significantly lower levels of these cytokines in both serum and placental tissues compared to the STZ + vehicle group and the STZ + TD139 group (all *p*<0.05). Moreover, TD139 markedly attenuated the inflammatory response in STZ‐induced GDM mice, as evidenced by reduced levels of TNF‐α, IL‐1β, IL‐6, and MCP‐1 (all *p*<0.05). No significant differences were observed in the expression of these cytokines between the Control + vehicle group and the Control + TD139 group (all *p*>0.05).

### 3.5 TD139 suppresses ERK/JNK/p38 signaling in placental tissues of STZ‐induced GDM mice *in vivo*

**FIGURE 5:**
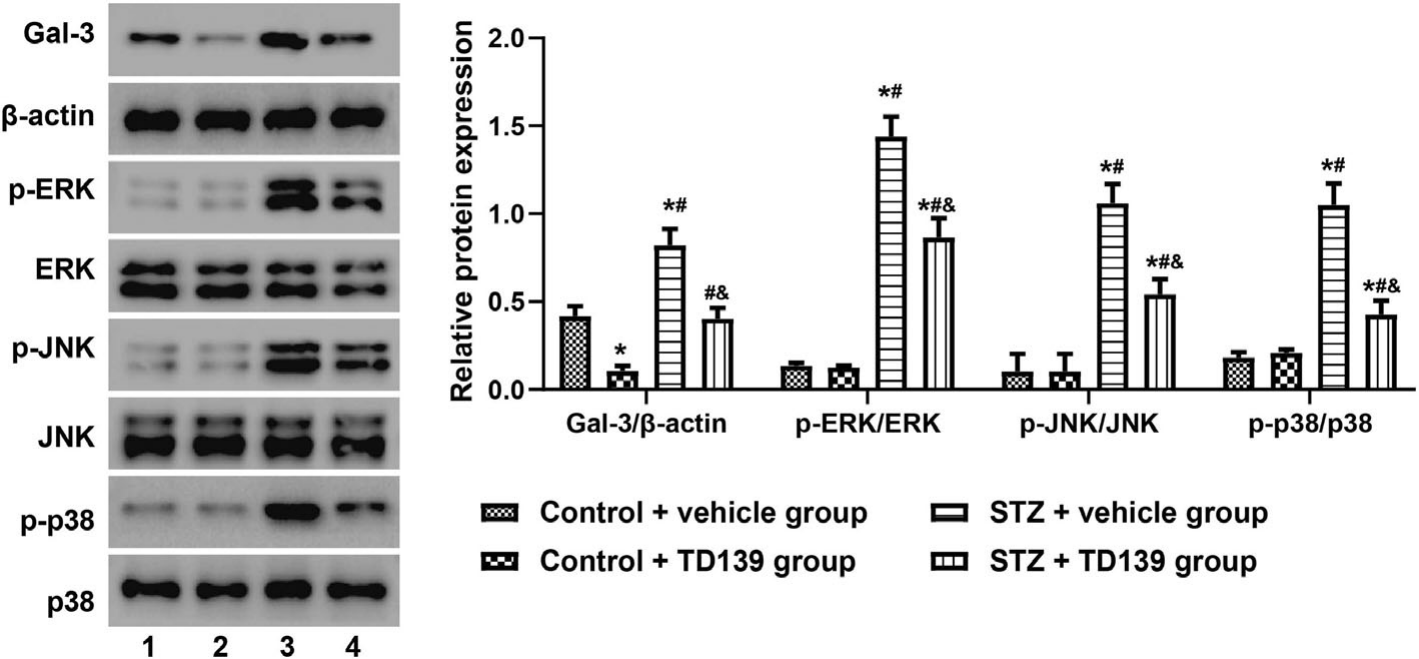
TD139 Suppresses ERK/JNK/p38 signaling in placental tissues of streptozotocin (STZ)‐induced gestational diabetes mellitus (GDM) mice in vivo. Western blotting was used to detect Galectin‐3 (Gal‐3) expression and the levels of phosphorylated extracellular signal‐regulated kinase (p‐ERK/ERK), c‐Jun N‐terminal kinase (p‐JNK/JNK), and p38 mMitogen‐activated protein kinase (p‐p38/p38) in the placental tissues after the birth of the pups across different groups (*n* = 6 for each group). 1, Control + vehicle group; 2, Control + TD139 group; 3, STZ + vehicle group; 4, STZ + vehicle group; * indicates *p*<0.05 compared to the Control + vehicle group; # indicates *p*<0.05 compared to the Control + TD139 group; & indicates *p*<0.05 compared to the STZ + vehicle group.

As shown in Figure 5, animal experiments revealed that Gal‐3 expression at the protein level was significantly increased in the placental tissues of STZ‐induced GDM mice (*p*<0.05). TD139 reduced Gal‐3 expression in the placental tissues of both control mice and GDM mice, with lower levels observed in the control mice (*p*<0.05). ERK/JNK/p38 signaling was activated in the placental tissues of STZ‐induced GDM mice compared to the Control + vehicle group and the Control + TD139 group (all *p*<0.05). In the STZ + TD139 group, ERK/JNK/p38 signaling in the placental tissues was inhibited compared to the STZ + vehicle group (all *p*<0.05). There were no significant differences in the levels of phosphorylated ERK (p‐ERK/ERK), JNK (p‐JNK/JNK), and p38 (p‐p38/p38) in the placental tissues of the Control + vehicle group and the Control + TD139 group (all *p*>0.05).

## 4 DISCUSSION

TNF‐α is a pro‐inflammatory cytokine implicated in the pathogenesis of insulin resistance and is known to induce inflammation in placental tissue.^24^ Human placental tissue samples were stimulated with TNF‐α *in vitro* to create a GDM‐like environment following established protocols from previous studies.^25^, ^26^ Our key findings demonstrate that TNF‐α stimulation in placental tissue significantly increased Gal‐3 expression, highlighting the potential of Gal‐3 inhibition as a therapeutic target for GDM.

Clinically, elevated Gal‐3 levels were observed in GDM maternal blood and placental tissue,^16^ which aligns with our findings of increased Gal‐3 levels in STZ‐induced GDM mouse placental tissue. Furthermore, circulating Gal‐3 levels are elevated in GDM^15^ and positively associated with progesterone levels and insulin resistance, suggesting a potential role of Gal‐3 in mediating GDM pathogenesis through insulin resistance and its interaction with progesterone.^14^ Interestingly, a previous study unveiled increased Gal‐3 mRNA expression induced by TNF‐α in knee osteoarthritis synoviocytes.^27^ However, TNF‐α stimulation promoted the production of psoriasis‐related inflammatory mediators along with the inhibition of Gal‐3 expression in keratinocytes.^28^ Additionally, no statistically significant correlation was observed between serum Gal‐3 levels and serum TNF‐α levels in psoriasis patients.^29^ Furthermore, treatment with TNF‐α had no effect on Gal‐3 mRNA expression in cytotrophoblasts and syncytiotrophoblasts.^30^ These diverse effects of Gal‐3 in TNF‐α‐induced inflammatory conditions indicate a complex regulatory mechanism that varies across different tissues and disease contexts.

In various preclinical models, TD139 has shown promise, alleviating neuroinflammatory damage in experimental subarachnoid hemorrhage^31^ and mitigating NKT‐cell‐dependent hepatitis by reducing pro‐inflammatory cytokine production and liver inflammation.^32^ Similarly, in experimental autoimmune uveitis mice, TD139 effectively alleviated uveitis symptoms and suppressed proinflammatory factors in microglia, suggesting its potential as a therapeutic intervention for autoimmune uveitis.^33^ More importantly, Gal‐3 deficiency or TD139 treatment attenuates cytokine‐triggered apoptosis of beta cells by modulating the mitochondrial apoptotic pathway, suggesting a pivotal role of endogenous Gal‐3 in mediating beta cell apoptosis in the inflammatory milieu associated with diabetes pathogenesis.^6^ In our study, TNF‐α stimulation led to increased pro‐inflammatory cytokines such as IL‐1β, IL‐6, and MCP‐1 expression, which was significantly reduced by TD139 treatment. Additionally, TD139 significantly lowered the levels of inflammatory cytokines (IL‐1β, IL‐6, and MCP‐1) in both serum and placental tissues in GDM mice. These cytokines are key mediators of inflammation and play pivotal roles in the pathogenesis of GDM by impairing placental function.^34^ The observed decrease in cytokine levels with TD139 treatment indicates its potential to modulate inflammatory responses, thereby improving metabolic and placental health in GDM.

In our *in vivo* experiments, STZ‐induced GDM mice exhibited significantly elevated glucose levels, reduced body weight, increased food intake (indicative of polyphagia), and higher water consumption (indicative of polydipsia). These symptoms are hallmark features of diabetes, reflecting severe metabolic disturbances.^21^ Gal‐3 levels correlate with insulin, glucose, insulin resistance, and dyslipidemia in women with polycystic ovary syndrome.^35^ Petrovic I et al. found that overexpression of Gal‐3 in pancreatic beta cells exacerbates β‐cell apoptosis, islet inflammation, oxidative stress, and dysregulates glucose metabolism, contributing to the development of obesity‐induced T2DM in mice.^36^ However, studies in humans have shown that increased Gal‐3 concentration is associated with unbridled glucose homeostasis.^37^ Baek et al. recently reported that Gal‐3 knockout mice had significantly lower body weight, reduced epididymal white adipose tissue, and higher expression of adipose triacylglycerol lipase, the rate‐limiting enzyme in fat cell lipolysis, compared to wild‐type animals after 12 weeks on a high‐fat diet.^38^ Conversely, obese mice with Gal‐3 ablation have increased body weight, fasting blood glucose, insulin levels, and markers of systemic inflammation compared with diet‐matched wild‐type animals.^39^ In our study, TD139 administration mitigated the adverse metabolic changes induced by STZ in GDM mice. This was evidenced by reduced glucose levels, increased body weight, decreased food and water consumption, and enhanced pregnancy outcomes. The underlying mechanisms for these effects likely involve the inhibition of Gal‐3, which reduces inflammation and improves insulin sensitivity, thereby ameliorating the metabolic disturbances associated with GDM. These findings suggest that targeting Gal‐3 may offer a therapeutic strategy to manage metabolic dysregulation in GDM and potentially other metabolic disorders.

The ERK/JNK/p38 signaling pathway is integral to cellular responses to stress, inflammation, and other stimuli, and its activation has been linked to various pathological conditions, including diabetes and its complications.^7^ Gal‐3 knockdown attenuates cardiac ischemia–reperfusion injury by interacting with Bcl‐2, modulating mitochondrial function, and inhibiting inflammation response in cardiomyocytes, possibly through regulation of the JNK pathway.^10^ Gal‐3 regulates ultraviolet‐induced skin inflammation by modulating the production of inflammatory cytokines, reactive oxygen species, and phosphorylation of p38 in human keratinocytes, while its knockout attenuates erythema, tissue inflammation, and expression of active IL‐1β and COX2 in mice.^8^ Gal‐3 binding protein secreted by neuroblastoma cells stimulates IL‐6 production in bone marrow mesenchymal stem cells via a Gal‐3BP/Gal‐3/Ras/MEK/ERK signaling pathway.^9^ In our study, TNF‐α stimulation activated the ERK/JNK/p38 pathway in human placental tissues, as evidenced by increased levels of phosphorylated ERK (p‐ERK), JNK (p‐JNK), and p38 (p‐p38). This activation was significantly suppressed by TD139 treatment, indicating that TD139 effectively inhibits this pathway. The inhibition of the ERK/JNK/p38 pathway by TD139 is particularly relevant in the context of GDM, where enhanced activation of these kinases can lead to increased production of inflammatory cytokines and subsequent placental dysfunction. By mitigating the activation of this pathway, TD139 helps to reduce the inflammatory milieu within the placenta, potentially leading to better pregnancy outcomes.

While our study provides significant insights into the mechanisms of TD139 action in GDM, several limitations need to be acknowledged. First, we used TNF‐α to induce a GDM‐like environment in human placental tissue samples. However, we recognize that while this *in vitro* model aimed to mimic aspects of GDM pathology, it only partially captures the complexity of the disease process. GDM development involves various factors such as hormonal fluctuations, placental functional changes, and the interplay of genetic and metabolic factors in the host. For future investigations, exploring additional *in vitro* GDM models could further validate our experimental outcomes. Second, the study was conducted using an STZ‐induced GDM mouse model, which, although useful, may not fully replicate the complexity of human GDM. Future studies should aim to validate these findings in clinical settings with human subjects. Third, while we focused on the ERK/JNK/p38 signaling pathway, GDM is a multifactorial condition involving multiple signaling cascades. It would be beneficial to explore the effects of TD139 on other relevant pathways to gain a comprehensive understanding of its therapeutic potential. Finally, the long‐term safety and efficacy of TD139 need to be evaluated to ensure its suitability for use in pregnant women. The potential effects on fetal development and postnatal health are critical factors that require thorough investigation.

## 5 CONCLUSION

Our study highlights the significant anti‐inflammatory and metabolic benefits of TD139 in GDM, mediated through the ERK/JNK/p38 signaling pathway. These findings pave the way for further research and potential clinical application of TD139 in managing GDM, ultimately aiming to improve maternal and fetal health outcomes.

## CONFLICT OF INTEREST STATEMENT

All authors declare no conflict of interest.
